# Economic Burden of Healthcare Services on Cancer Survivors in Bangladesh

**DOI:** 10.1002/cnr2.2144

**Published:** 2024-08-09

**Authors:** Md. Shahjalal, Padam Kanta Dahal, Md. Parvez Mosharaf, Mohammad Morshad Alam, Mohammad Delwer Hossain Hawlader, Rashidul Alam Mahumud

**Affiliations:** ^1^ Department of Public Health North South University Dhaka Bangladesh; ^2^ Research Rats Dhaka Bangladesh; ^3^ School of Health, Medical and Applied Sciences Central Queensland University Sydney New South Wales Australia; ^4^ School of Business and Centre for Health Research University of Southern Queensland Toowoomba Queensland Australia; ^5^ Health Economics and Health Technology Assessment Unit, NHMRC Clinical Trials Centre, Faculty of Medicine and Health The University of Sydney Camperdown New South Wales Australia

**Keywords:** Bangladesh, burden of cancer, cancer, economic, healthcare cost

## Abstract

**Background:**

Cancer is a critical public health issue that imposes a considerable economic burden, especially in low‐resource countries. In Bangladesh, there has been a noticeable lack of research focusing on the economic burden associated with cancer. **Aims:** This study aimed to examine the economic burden of cancer care and the contributing factors.

**Methods:**

This cross‐sectional study included 623 cancer patients. Data were collected between January and May 2022. The magnitude of the economic burden (no burden to extreme burden) was the outcome variable. A logistic regression model was performed to determine the associated factors of the economic burden of cancer.

**Results:**

Overall, 34% of cancer survivors experienced extreme economic burden due to treatment costs. Patients with prostate (relative risk ratio, RRR = 23.24; 95% confidence interval, CI: 1.97, 273.70), bone (RRR = 5.85; 95% CI: 1.10, 31.04), and liver cancer (RRR = 4.94; 95% CI: 1.29, 18.9) reported significantly higher extreme economic burden compared to patients with other cancers. The economic burden was significantly higher for patients diagnosed with Stage III (RRR = 38.69; 95% CI: 6.17, 242.72) and Stage IV (RRR = 24.74; 95% CI: 3.22, 190.11) compared to Stage 0. Patients from low‐income households suffered from nine times more extreme burden (RRR = 8.85; 95% CI: 4.05, 19.36) compared with those from high‐income households.

**Conclusion:**

Our study found a disproportionately high economic burden among patients with cancer, across disease sites, stages, and income quintiles. The burden was significantly higher among patients with prostate, bone, and liver cancer, and those diagnosed with advanced stage. The findings underscore the importance of early cancer detection before metastasis which may lead to more efficient treatment, avoid disease progression, lower disease management costs, and better health outcomes. Patients from low‐income households experience an extreme economic burden due to cancer, highlighting the need for affordable healthcare services, financial support, and healthcare subsidies.

## 1 Introduction

Cancer is a leading cause of the burden of the global disease that accounts for a large portion of global morbidity and mortality [1]. According to the latest GLOBOCAN report, cancer caused 9.7 million deaths in 2020 worldwide [2]. The cancer mortality rate is increasing in low‐ and middle‐income countries (LMICs), and it is anticipated that approximately two‐thirds of all cancer‐related fatalities will occur in these regions by 2030 [3].

The number of cancer survivors continues to grow globally due to earlier cancer diagnoses and progressively more complex treatments [4, 5]. Cancer survivors often need continuation of care and that care may last for an extended period [5]. Studies highlight that long‐term cancer treatment, posttreatment care, and patient monitoring create a heavy economic burden on patients, their families, society, and the healthcare sector [6, 7, 8, 9]. The economic impact of cancer may be compounded by factors such as cancer type, poor prognosis, treatment modalities, presence of comorbidities, cost of care and socioeconomic status, and healthcare insurance policy [7, 8, 9, 10]. In recent years, cancer treatment costs have increased remarkably, by two to threefold for some cancers, especially at the later stages of the disease [11, 12]. Along with treatment‐related burdens, cancer imposes a heavy burden on the economy resulting in loss of productivity, increased unemployed people, reduced workforces, and capital expenditure cuts [7, 10].

The published literature on the economic burden of cancer has mainly focused on economically advanced nations, highlighting the significant adverse impact of cancer on the national economy [13, 14, 15, 16]. In the United States, patients faced a $21.09 billion economic burden associated with cancer care in 2019, comprising $16.22 billion in out‐of‐pocket (OOP) expenses [14]. In Europe, the total cost of cancer was $294 billion in 2018 [15]. Similarly, some studies, including those conducted in LMICs, reported a significant economic impact on cancer [17, 18, 19]. In India, the total direct OOP cost of cancer treatment was estimated to be $4174 per patient per year [17]. In Nepal, on average, cancer patients spend $3252 on direct cancer care each year [18]. While OOP healthcare expenditure is a serious concern for LMICs, the increasing costs of cancer treatment are placing a strain on public healthcare budgets [20]. As a result, costs directly associated with healthcare services, such as OOP expenditure, and indirect costs like lost income and productivity through increasing cancer incidence and premature cancer death combine to create a catastrophic economic burden in LMICs [17, 18, 19, 20].

According to the latest GLOBOCAN statistics, an estimated to be around 167 256 new cancer cases and 116 598 mortalities in Bangladesh in 2022 [21]. In addition, the report shows an overall cancer incidence rate of 105.6 per 100 000 persons, with a mortality rate of 74.7 per 100 000 persons. As a result of such a high incidence and mortality rate, cancer may have a significant economic impact, where the country's people have the least personal resources to cover these costs [22]. In Bangladesh, OOP expenditure is the primary healthcare payment method, resulting in around 16% of households facing high health expenses which plunge many households into poverty [22]. In turn, cancer patients and their families may have to choose between treatment and basic necessities due to healthcare costs. Hence, it is essential to understand and assess the economic burden to mitigate cancer impact. In Bangladesh, the authors did not find any studies that comprehensively addressed the economic burden of cancer. Therefore, it has become an urgent and practical research topic to explore the economic burden of cancer and its influencing factors that affect the care of cancer patients and provide scientific evidence to healthcare policymakers. As a result, the present study aimed to examine the economic burden of cancer and its contributing factors.

## 2 Methods

### 2.1 Study Design and Settings

This study site was conducted at two largest tertiary care specialized cancer hospitals in Dhaka, Bangladesh. Dhaka is the capital and largest city of Bangladesh with a population of over 15 million. This South Asian country ranks as the eighth‐most populous nation worldwide with 171.2 million population in 2022 [23]. The study hospitals were the National Institute of Cancer Research & Hospital and Ahsania Mission Cancer & General Hospital. Data were collected between January and May 2022.

### 2.2 Participants and Survey Procedures

The study population was patients diagnosed with cancer attending these facilities for outpatient services. We included patients diagnosed with the first primary tumor site where they were receiving treatments for a specific cancer tumor site at the facility. If any patient experienced tumors in multiple sites, we considered the first primary tumor site. Data were collected by the trained research team, where necessary training for data collection, report building and preserving neutrality, and informed them on ethical issues for cancer patients were provided. Further, a pilot survey was conducted to explore the ability to understand useful techniques and possible challenges during interviews. Patients attending study hospitals for treatment were invited voluntarily to participate in the survey. We excluded patients with severe mental health issues, unable to answer the question due to severe illnesses, and refusal to consent. A face‐to‐face interview was performed one by one to ensure their privacy. Approximately 800 cancer patients were invited to participate in the study, while 645 patients were interviewed based on the inclusion criteria (response rate: 80.6%). We selected a total of 623 data items for analysis after eliminating incomplete and insufficient quality information (Appendix 1).

### 2.3 Outcome Measure

Participants were asked about the economic burden of cancer, “For this treatment what type of economic burden your family has faced?” The burden of cancer was assessed based on this question. The response was categorized as “0 = no burden and/or limited burden,” “1 = severe burden,” and “2 = extreme burden.”

### 2.4 Clinical Characteristics

We considered several clinical characteristics, including cancer site and stage. Type of cancer site and stage‐related data were collected from the patient's pathological diagnostic reports or received current relevant treatment. The cancer stage was categorized as Stage 0, I, II, III, and IV. The most reported diagnosed cancer sites were categorized as oral, breast, blood, pancreas, liver, lung, brain, throat, cervical, and other cancer sites (e.g., eye, skin, bone, penile, ovarian).

### 2.5 Covariates

In this study, the socioeconomic model was used to explain the economic burden of cancer on individuals, and explanatory variables were selected based on this model and examined for possible confounding variables [6, 24]. This study also considered individual‐level factors like demographic characteristics which included gender, age, body mass index (BMI), marital status, education, residence, household size, occupational status, and monthly household income were considered as predisposing factors in the analysis. Patient's gender was classified as male or female, and age was grouped as 18–35 years, 36–45 years, 46–64 years, or >64 years. The BMI was classified as underweight: <18.49 kg/m^2^, healthy weight: 18.50–24.99 kg/m^2^, overweight: 25–29.99 kg/m^2^, or obese: ≥30 kg/m^2^. Marital status was defined as single/never married, married, or widowed/divorced. Educational background was defined as no education, primary: 1–5 years, secondary: 6–10 years, higher secondary: 10–12 years, or tertiary: over 12 years of schooling. Location of residence was dichotomized as either urban or rural. Household size was classified as <4 members, 4–5 members, or ≥ 6 members. Occupational status was defined as unemployed, employed, business, informal workers (e.g., street vendors, wage laborers), housewife, student, or other occupations. The monthly household income quintile was divided into five strata: poorest (Q_1_: lowest 20%), poorer (Q_2_), middle (Q_3_), richer (Q_4_), and richest (Q_5_: top 20%).

### 2.6 Statistical Analysis

Descriptive statistics were expressed in frequency and percentages (with 95% confidence interval, CI). For the univariate and multivariable analyses, we chose the estimation approach based on the nature of the outcome variables in each model. Multilevel‐ordered logistic regression analysis was performed to identify the potential factors associated with economic burden. The regression model was selected based on the nature of the outcome variable and its distribution. The relative risk ratio (RRR) with 95% CI was used to measure the risk among the comparator groups. The explanatory variables were included in the adjusted model only if any label of the predictor was significant at ≤5% risk level in the unadjusted regression model. All analyses were performed using the statistical software STATA/SE‐15 (Stata Corp LP, College Station, TX, USA).

## 3 Results

### 3.1 Participant's Characteristic

The characteristics of the participants are presented in Table 1. Out of the 623 patients, 55% were female and 51% were aged between 46 and 64 years. A higher proportion of the patients (61%) had a normal weight. Most survivors were married (87%) and lived in rural communities (88%). Approximately, 64% of cancer survivors were not employed and 30% came from low‐income households. The majority of patients were affected by breast (17%), oral (11%), and lung cancer (10%). Similarly, the majority of patients had Stage I (43%) and Stage II cancer (32%).

**TABLE 1:** Patients' basic characteristics (*n* = 623).

Patients' characteristics	*n* (%)	95% CI
Cancer type		
Oral	67 (10.75)	(9.00, 13.00)
Brest	109 (17.50)	(15.00, 21.00)
Lung	66 (10.59)	(8.00, 13.00)
Blood	19 (3.05)	(2.00, 5.00)
Gallbladder	9 (1.44)	(1.00, 3.00)
Pancreas	13 (2.09)	(1.00, 4.00)
Liver	21 (3.37)	(2.00, 5.00)
Kidney	7 (1.12)	(1.00, 2.00)
Colorectal	31 (4.98)	(4.00, 7.00)
Prostate	11 (1.77)	(1.00, 3.00)
Urinary tract	9 (1.44)	(1.00, 3.00)
Cervical	86 (13.80)	(11.00, 17.00)
Brain	13 (2.09)	(1.00, 4.00)
Gastric	9 (1.44)	(1.00, 3.00)
Bone	16 (2.57)	(2.00, 4.00)
Throat	21 (3.37)	(2.00, 5.00)
Esophageal	9 (1.44)	(1.00, 3.00)
Others	107 (17.17)	(14.00, 20.00)
Cancer stages		
Stage 0	20 (3.21)	(2.00, 5.00)
Stage I	265 (42.54)	(39.00, 46.00)
Stage II	202 (32.42)	(29.00, 36.00)
Stage IV	33 (5.30)	(4.00, 7.00)
Age, years		
<18	16 (2.57)	(1.58, 4.16)
18–35	84 (13.48)	(11.01, 16.41)
36–45	130 (20.87)	(17.85, 24.25)
46–65	314 (50.40)	(39.17, 46.95)
>65	79 (12.68)	(17.10, 23.40)
Gender		
Male	283 (45.43)	(41.54, 49.37)
Female	340 (54.57)	(50.63, 58.46)
Marital status		
Single or never married	40 (6.42)	(4.74, 8.64)
Married	537 (86.20)	(83.25, 88.69)
Divorced or separated or widowed	46 (7.38)	(5.57, 9.73)
Education		
No education	287 (46.23)	(42.33, 50.17)
Primary	159 (25.52)	(22.24, 29.10)
Secondary	103 (16.53)	(13.81, 19.67)
Higher secondary	45 (7.22)	(5.43, 9.55)
Tertiary	28 (4.49)	(3.12, 6.44)
BMI group		
Underweight	106 (17.01)	(14.26, 20.18)
Healthy weight	380 (61.00)	(57.09, 64.76)
Overweight	92 (14.77)	(12.19, 17.78)
Obese	45 (7.22)	(5.43, 9.55)
Family size		
<4 members	76 (12.20)	(9.85, 15.02)
4–5 members	311 (49.92)	(45.99, 53.85)
≥6 members	236 (37.88)	(34.14, 41.77)
Occupation		
Unemployed	142 (22.79)	(19.66, 26.26)
Employed	39 (6.26)	(4.60, 8.46)
Business	41 (6.58)	(4.88, 8.82)
Housewife	248 (39.81)	(36.02, 43.72)
Informal worker	41 (6.58)	(4.88, 8.82)
Students	26 (4.17)	(2.85, 6.06)
Other occupations	86 (13.80)	(11.31, 16.75)
Income quintile		
Q_1_ (20% lowest)	186 (29.86)	(30.87, 38.34)
Q_2_	168 (26.97)	(6.69, 11.15)
Q_3_	41 (6.58)	(15.45, 21.54)
Q_4_	120 (19.26)	(18.15, 24.58)
Q_5_ (20% highest)	108 (17.34)	(14.55, 20.52)
Residence		
Rural	546 (87.64)	(84.81, 90.01)
Urban	77 (12.36)	(9.99, 15.19)

### 3.2 Magnitude of Economic Burden of Cancer

The magnitude of the economic burden of cancer is presented in Table 2. Among patients who reported severe burden, 21% had breast cancer, 14% had cervical cancer, 10% had lung cancer, and 7% had oral cancer. Similarly, in case of extreme burden, 18% had breast cancer, 15% had oral cancer, 13% had cervical cancer, and 12% had lung cancer. Patients with advanced cancer had a greater severe and extreme economic burden as compared to those with early‐stage cancer. Approximately 1% of patients with Stage 0 cancer experienced extreme burden, and 5% at Stage IV.

**TABLE 2:** Magnitude of economic burden among cancer patients.

	Magnitude of economic burden of cancer		
	No/limited burden	Severe burden	Extreme burden
Variables	% (95% CI)	% (95% CI)	% (95% CI)
Cancer types			
Oral	10.77 (7.11, 15.98)	6.51 (3.89, 10.72)	15.02 (10.81, 20.50)
Breast	13.33 (9.22, 18.89)	20.93 (15.99, 26.91)	17.84 (13.24, 23.60)
Lung	10.26 (6.70, 15.39)	10.23 (6.82, 15.08)	11.27 (7.65, 16.29)
Blood	4.10 (2.06, 8.01)	2.79 (1.25, 6.09)	2.35 (0.98, 5.53)
Gallbladder	2.05 (0.77, 5.36)	1.40 (0.45, 4.26)	0.94 (0.23, 3.70)
Pancreatic	4.10 (2.06, 8.01)	0.47 (0.06, 3.25)	1.88 (0.70, 4.92)
Liver	2.56 (1.07, 6.03)	2.79 (1.25, 6.09)	4.69 (2.54, 8.53)
Kidney	2.05 (0.77, 5.36)	0.93 (0.23, 3.66)	0.47 (0.07, 3.28)
Colorectal	3.08 (1.38, 6.70)	5.12 (2.85, 9.02)	6.57 (3.92, 10.81)
Prostate	0.51 (0.07, 3.58)	1.86 (0.70, 4.87)	2.82 (1.27, 6.15)
Urinary tract	1.54 (0.49, 4.68)	2.33 (0.97, 5.48)	0.47 (0.07, 3.28)
Cervical	14.87 (10.52, 20.61)	13.49 (9.52, 18.76)	13.15 (9.22, 18.41)
Brain	2.05 (0.77, 5.36)	1.40 (0.45, 4.26)	2.82 (1.27, 6.15)
Gastric	2.05 (0.77, 5.36)	0.93 (0.23, 3.66)	1.41 (0.45, 4.29)
Bone	1.54 (0.49, 4.68)	1.40 (0.45, 4.26)	1.40 (0.45, 4.26)
Throat	4.10 (2.06, 8.01)	4.65 (2.51, 8.45)	1.41 (0.45, 4.29)
Esophageal	0.51 (0.07, 3.58)	2.79 (1.25, 6.09)	0.94 (0.23, 3.70)
Other cancers	20.51 (15.40, 26.79)	20 (15.16, 25.91)	11.27 (7.65, 16.29)
Cancer stages			
Stage 0	6.67 (3.90, 11.17)	2.33 (0.97, 5.48)	0.94 (0.23, 3.70)
Stage I	47.18 (40.24, 54.23)	41.86 (35.42, 48.59)	38.97 (32.62, 45.71)
Stage II	30.26 (24.19, 37.09)	32.09 (26.17, 38.65)	34.74 (28.63, 41.41)
Stage III	10.77 (7.11, 15.98)	17.67 (13.12, 23.39)	20.66 (15.72, 26.65)
Stage IV	5.13 (2.77, 9.29)	6.05 (3.53, 10.16)	4.69 (2.54, 8.53)
Income quintile			
Q_1_ (20% lowest)	23.59 (18.13, 30.09)	30.23 (24.44, 36.73)	48.83 (42.15, 55.55)
Q_2_	6.15 (3.52, 10.55)	8.84 (5.70, 13.46)	10.8 (7.27, 15.75)
Q_3_	16.92 (12.27, 22.88)	20 (15.16, 25.91)	17.84 (13.24, 23.60)
Q_4_	23.59 (18.13, 30.09)	26.51 (21.02, 32.84)	13.62 (9.61, 18.93)
Q_5_ (20% highest)	29.74 (23.72, 36.56)	14.42 (10.31, 19.80)	8.92 (5.75, 13.58)
Occupational status			
Unemployed	15.38 (10.95, 21.18)	23.26 (18.07, 29.40)	29.11 (23.38, 35.59)
Employed	7.18 (4.29, 11.78)	9.30 (6.07, 14.00)	2.35 (0.98, 5.53)
Business	8.21 (5.08, 13)	7.44 (4.60, 11.82)	4.23 (2.21, 7.94)
Housewife	38.97 (32.36, 46.03)	42.79 (36.32, 49.52)	37.56 (31.29, 44.28)
Informal worker	4.10 (2.06, 8.01)	5.58 (3.19, 9.59)	9.86 (6.50, 14.67)
Students	7.18 (4.29, 11.78)	1.40 (0.45, 4.26)	4.23 (2.21, 7.94)
Other occupations	18.97 (14.05, 25.12)	10.23 (6.82, 15.08)	12.68 (8.82, 17.88)
Age in years			
<18	5.13 (2.77, 9.29)	1.40 (0.45, 4.26)	1.41 (0.45, 4.29)
18–35	8.72 (5.48, 13.60)	13.95 (9.92, 19.28)	17.37 (12.84, 23.08)
36–45	20.51 (15.40, 26.79)	16.28 (11.91, 21.86)	25.82 (20.37, 32.15)
46–65	36.41 (29.93, 43.42)	50.23 (43.56, 56.90)	41.78 (35.32, 48.55)
>65	29.23 (23.25, 36.03)	18.14 (13.53, 23.89)	13.62 (9.61, 18.93)
Gender			
Male	47.18 (40.24, 54.23)	44.19 (37.66, 50.92)	45.07 (38.49, 51.83)
Female	52.82 (45.77, 59.76)	55.81 (49.08, 62.34)	54.93 (48.17, 61.51)
Education			
No education	36.92 (30.41, 43.95)	46.05 (39.47, 52.77)	54.93 (48.17, 61.51)
Primary	27.18 (21.38, 33.88)	26.98 (21.45, 33.33)	22.54 (17.4, 28.66)
Secondary	18.97 (14.05, 25.12)	16.74 (12.31, 22.37)	14.08 (10.01, 19.46)
Higher secondary	10.77 (7.11, 15.98)	5.58 (3.19, 9.59)	5.63 (3.22, 9.68)
Tertiary	6.15 (3.52, 10.55)	4.65 (2.51, 8.45)	2.82 (1.27, 6.15)
Marital status			
Single/unmarried	8.21 (5.08, 13.00)	3.72 (1.87, 7.28)	7.51 (4.64, 11.93)
Married	84.10 (78.25, 88.61)	92.09 (87.63, 95.04)	82.16 (76.40, 86.76)
Divorced/widowed	7.69 (4.68, 12.39)	4.19 (2.19, 7.87)	10.33 (6.89, 15.21)
Family size			
<4 members	11.28 (7.53, 16.57)	10.23 (6.82, 15.08)	15.02 (10.81, 20.50)
4–5 members	53.85 (46.79, 60.75)	51.63 (44.93, 58.27)	44.60 (38.03, 51.36)
≥6 members	34.87 (28.49, 41.85)	38.14 (31.86, 44.84)	40.38 (33.97, 47.13)
BMI			
Underweight	14.36 (10.08, 20.04)	15.35 (11.11, 20.83)	21.13 (16.14, 27.15)
Normal weight	60.51 (53.46, 67.16)	63.26 (56.58, 69.46)	59.15 (52.4, 65.58)
Overweight	16.92 (12.27, 22.88)	14.42 (10.31, 19.80)	13.15 (9.22, 18.41)
Obese	8.21 (5.08, 13.00)	6.98 (4.24, 11.27)	6.57 (3.92, 10.81)
Residential status			
Rural	83.59 (77.68, 88.17)	92.09 (87.63, 95.04)	86.85 (81.59, 90.78)
Urban	16.41 (11.83, 22.32)	7.91 (4.96, 12.37)	13.15 (9.22, 18.41)

Abbreviations: BMI, body mass index; CI, confidence interval.

The economic burden of cancer was higher for patients from the low‐income quintile, with 31% reporting severe and 49% reporting extreme burdens. In contrast, patients from high‐income quintiles experienced lower levels of severe (15%) and extreme (9%) burden compared with low‐income families. Housewives (severe = 43% and extreme = 38%) and unemployed patients (severe = 24% and extreme = 30%) had the highest burden. Two‐thirds of rural patients experienced severe and extreme economic burdens (Table 2).

Appendix 2 presents the results in gender terms, which shows patients with lung cancer in males and patients with breast cancer in females experienced a higher severe and extreme economic burden (Table A1).

### 3.3 Factors Influencing Economic Burden of Cancer

The factors associated with the economic burden on cancer patients are highlighted in Table 3. Patients diagnosed with specific types of cancer experienced a higher extreme economic burden due to cancer treatment. This includes prostate cancer (RRR = 23.24, 95% CI: 1.97, 273.701), bone cancer (RRR = 5.85; 95% CI: 1.10, 31.04), and liver cancer (RRR = 4.94, 95% CI: 1.29, 18.9).

**TABLE 3:** Associated factors of economic burden among cancer patients.

Variables	Severe burden vs. No/limited burden		Extreme burden vs. No/limited burden	
	Unadjusted RRR (95% CI)	Adjusted RRR (95% CI)	Unadjusted RRR (95% CI)	Adjusted RRR (95% CI)
Cancer type (ref = other types)				
Oral	0.62 (0.28, 1.38)	0.49 (0.20, 1.22)	2.54 (1.20, 5.36)	2.60 (1.03, 6.53)*
Breast	1.61 (0.84, 3.07)	1.45 (0.64, 3.29)	2.44 (1.20, 4.96)	2.47 (0.97, 6.34)
Lung	1.02 (0.49, 2.15)	0.93 (0.39, 2.18)	2.00 (0.92, 4.36)	1.68 (0.65, 4.35)
Blood	0.70 (0.22, 2.19)	1.19 (0.28, 5.08)	1.04 (0.31, 3.55)	2.03 (0.42, 9.69)
Gallbladder	0.70 (0.15, 3.31)	0.63 (0.11, 3.53)	0.83 (0.14, 4.90)	1.08 (0.15, 7.66)
Pancreatic	0.12 (0.01, 0.97)	0.12 (0.01, 1.17)	0.83 (0.23, 3.07)	0.74 (0.13, 4.05)
Liver	1.12 (0.32, 3.95)	0.96 (0.25, 3.70)	3.33 (1.02, 10.92)	4.94 (1.29, 18.9)*
Kidney	0.47 (0.08, 2.68)	0.29 (0.04, 1.98)	0.42 (0.04, 3.95)	0.29 (0.03, 3.29)
Colorectal	1.71 (0.58, 5.04)	1.00 (0.29, 3.47)	3.89 (1.32, 11.47)	1.68 (0.45, 6.18)
Prostate	3.72 (0.4, 34.72)	4.54 (0.41, 50.56)	10.0 (1.13, 88.17)	23.24 (1.97, 273.70)**
Urinary tract	1.55 (0.35, 6.91)	1.45 (0.27, 7.81)	0.56 (0.05, 5.65)	0.59 (0.04, 7.95)
Cervical	0.93 (0.48, 1.82)	0.75 (0.32, 1.75)	1.61 (0.78, 3.32)	1.55 (0.59, 4.03)
Brain	0.70 (0.15, 3.31)	0.93 (0.15, 5.73)	2.50 (0.64, 9.77)	2.92 (0.52, 16.28)
Gastric	0.47 (0.08, 2.68)	0.57 (0.08, 3.88)	1.25 (0.26, 6.07)	1.30 (0.19, 8.96)
Bone	0.93 (0.18, 4.88)	1.20 (0.20, 7.41)	5.56 (1.39, 22.21)	5.85 (1.10, 31.04)
Throat	1.16 (0.42, 3.24)	0.70 (0.21, 2.34)	0.63 (0.15, 2.59)	0.36 (0.07, 1.87)
Esophageal	5.58 (0.64, 48.41)	2.55 (0.26, 25.06)	3.33 (0.29, 38.75)	1.56 (0.11, 22.41)
Cancer stage (ref = stage 0)				
Stage I	2.54 (0.87, 7.43)	2.62 (0.74, 9.33)	5.86 (1.29, 26.76)	8.3 (1.45, 47.64)
Stage II	3.04 (1.02, 9.03)	3.27 (0.89, 12.01)	8.15 (1.77, 37.56)	12 (2.05, 70.27)
Stage III	4.70 (1.47, 15.02)	6.61 (1.65, 26.53)**	13.62 (2.81, 65.91)	38.69 (6.17, 242.72)**
Stage IV	3.38 (0.90, 12.66)	7.34 (1.52, 35.54)**	6.50 (1.16, 36.58)	24.74 (3.22, 190.11)**
Income quintile (ref = Q_5_; richest)				
Q_1_	2.64 (1.48, 4.71)	3.35 (1.66, 6.78)**	6.90 (3.70, 12.88)	8.85 (4.05, 19.36)**
Q_2_	2.96 (1.27, 6.89)	5.18 (1.93, 13.85)**	5.85 (2.45, 13.95)	12.31 (4.29, 35.36)**
Q_3_	2.44 (1.30, 4.57)	2.91 (1.40, 6.07)**	3.52 (1.75, 7.06)	4.52 (1.95, 10.47)**
Q_4_	2.32 (1.29, 4.16)	2.41 (1.24, 4.67)**	1.92 (0.96, 3.86)	2.17 (0.97, 4.86)
Occupation (ref = employed)				
Unemployed	1.17 (0.51, 2.65)	0.92 (0.32, 2.62)	5.79 (1.91, 17.56)	4.29 (1.05, 17.44)**
Business	0.70 (0.26, 1.85)	0.55 (0.16, 1.85)	1.58 (0.43, 5.82)	1.81 (0.36, 9.15)
Housewife	0.85 (0.40, 1.79)	0.59 (0.20, 1.72)	2.95 (1.01, 8.58)	1.35 (0.32, 5.69)
Informal worker	1.05 (0.34, 3.24)	0.57 (0.14, 2.28)	7.35 (1.99, 27.13)	4.50 (0.83, 24.45)
Students	0.15 (0.04, 0.62)	0.10 (0.01, 0.73) *	1.80 (0.48, 6.74)	1.27 (0.15, 10.39)
Other occupations	0.42 (0.18, 0.99)	0.21 (0.07, 0.63)**	2.04 (0.66, 6.36)	0.79 (0.18, 3.45)
Age, years (ref = <18 years)				
18–35	5.88 (1.42, 24.36)	3.44 (0.39, 29.94)	7.25 (1.77, 29.78)	11.23 (1.45, 86.82)**
36–45	2.92 (0.74, 11.45)	1.19 (0.12, 12.01)	4.58 (1.18, 17.73)	5.38 (0.57, 50.68)
46–64	5.07 (1.35, 19.07)	2.10 (0.21, 20.68)	4.18 (1.11, 15.76)	3.73 (0.40, 34.65)
>64	2.28 (0.59, 8.82)	0.95 (0.09, 9.60)	1.70 (0.43, 6.64)	1.33 (0.14, 12.85)
Gender (ref = male)				
Female	1.13 (0.76, 1.67)	0.81 (0.35, 1.87)	1.09 (0.74, 1.61)	0.91 (0.39, 2.12)
BMI (ref = underweight)				
Normal weight	0.98 (0.56, 1.71)	0.79 (0.39, 1.58)	0.66 (0.39, 1.13)	0.58 (0.29, 1.18)
Overweight	0.80 (0.39, 1.61)	0.66 (0.27, 1.6)	0.53 (0.26, 1.05)	0.49 (0.19, 1.26)
Obese	0.80 (0.33, 1.89)	0.43 (0.15, 1.25)	0.54 (0.23, 1.28)	0.29 (0.1, 0.85)**
Education (ref = tertiary)				
No education	1.65 (0.68, 4.03)	1.25 (0.38, 4.07)	3.25 (1.17, 9.04)	2.40 (0.61, 9.45)
Primary	1.31 (0.52, 3.29)	1.01 (0.32, 3.22)	1.81 (0.63, 5.20)	1.43 (0.36, 5.62)
Secondary	1.17 (0.45, 3.04)	0.90 (0.27, 2.99)	1.62 (0.54, 4.83)	1.23 (0.3, 5.05)
Higher secondary	0.69 (0.23, 2.06)	0.43 (0.12, 1.57)	1.14 (0.34, 3.83)	0.70 (0.16, 3.15)
Marital status (ref = single or unmarried)				
Married	2.41 (1.01, 5.78)	0.91 (0.15, 5.33)	1.07 (0.52, 2.20)	0.95 (0.16, 5.45)
Divorced/widowed	1.20 (0.37, 3.92)	0.46 (0.06, 3.44)	1.47 (0.56, 3.81)	1.35 (0.19, 9.65)
Family size (ref = <4 members)				
4–5 members	1.06 (0.55, 2.02)	0.99 (0.46, 2.14)	0.62 (0.34, 1.14)	0.61 (0.28, 1.32)
≥6 members	1.21 (0.62, 2.36)	1.01 (0.45, 2.24)	0.87 (0.46, 1.63)	0.85 (0.38, 1.91)
Residence (ref = urban)				
Rural	2.29 (1.23, 4.27)	1.71 (0.81, 3.61)	1.30 (0.75, 2.25)	0.7 (0.33, 1.46)

Abbreviations: CI, confidence interval; ref, reference group; RRR, relative risk ratio.

*p‐value ≤ 0.05, **p‐value ≤ 0.01, and ***p‐value ≤ 0.001.

The study has revealed that the economic burden of cancer care was significantly higher for advanced cancer stages. Patients with Stage III cancer had a 39 times higher extreme economic burden (RRR = 38.69, 95% CI: 6.17, 242.72) compared with early stage (zero). Patients with Stage IV cancer had a 25 times greater extreme economic burden (RRR = 24.74, 95% CI: 3.22, 190.11) compared with early stage (zero). Additionally, the study found that the economic burden of cancer is significantly nine times higher among low‐income patients (RRR = 8.85, 95% CI: 4.05, 19.36) compared with the high‐income groups.

## 4 Discussion

This study aimed to estimate the economic burden of cancer care services in Bangladesh by analyzing patient‐reported data and identifying factors that influence the burden. Overall, 34% of patients experienced extreme economic burden due to cancer care services, whereas, this burden varies depending on the type and stage of cancer, with prostate, bone, and liver cancer showing a higher extreme burden. Our findings highlighted that advanced disease is significantly associated with higher economic burden. Moreover, individuals with a low socio‐economic background, unemployed, and female patients experienced a higher level of economic burden.

Our regression analysis suggests that patients with prostate cancer significantly had 20 times higher extreme economic burdens compared with other cancers. This result is similar to the findings of previous studies conducted in other countries [9, 25, 26]. For example, prostate cancer was one of the most economically burdensome cancers in the United States in 2020 [9]. Considering the overall economic burden on society, the prostate cancer burden increased faster than any other cancer in European countries [25]. However, slightly different findings from the other studies reported a high economic burden among patients with other cancers [6, 27]. These differences could be partly explained by the differences in cancer detection, treatment modalities and country‐specific healthcare systems. In recent years, the rate of prostate cancer has alarmingly increased among Asian men [26], including in Bangladesh, with 2335 new prostate cancer cases in 2022 [21]. As such keeping these statistics in mind, the authors stress increasing healthcare services to prevent and control prostate cancer in Bangladesh. Further, the authors also suggest awareness and early detection of prostate cancer among the vulnerable population as most men are diagnosed with advanced stages [28], which is highly linked to the high cost of care.

It is evident that bone is invaded in 60%–84% of all metastatic diseases [29] and patients with a history of radiation therapy have a higher risk of bone cancer in previously irradiated bone [30]. In our study, patients with bone cancer experienced a 6 times greater economic burden than those with other cancers. We can only speculate as to possible reasons for this finding. Bone cancer patients may have experienced high costs before detecting the cancer as laboratory studies are not particularly useful in diagnosis [31]. On the other hand, bone cancer patients may have previously received therapeutic treatment as previous exposure to ionizing radiation is considered a major risk factor for bone carcinoma [31]. When such a condition develops require intensive treatment in multiple modes to attain lasting disease control [30, 31] but these regimens have been associated with high cost [30]. Alarmingly, younger people are more likely to die from bone cancer than older people, which is highly concerning from an economic standpoint such as loss of productivity [30, 31]. Therefore, the authors urge policymakers to prioritize primordial and primary prevention of bone cancer focusing on their influencing factors to reduce the long‐term health and economic consequences.

Also, our results indicate that patients with liver cancer significantly had higher extreme economic burdens compared with other cancers. The burden of liver cancer is one of the highest in the world, and the burden is high in LMICs, including Bangladesh [32]. As such, this argument aligns with the previously conducted comparable studies in other countries [33, 34]. Liver cancer such as hepatocellular carcinoma, depends on the stage at which the cancer is diagnosed [35]. Unfortunately, liver cancer is often diagnosed at advanced stages, leading to significant challenges in treatment and causing low survival rates [33, 34, 35]. Currently in Bangladesh, early cancer detection is the biggest challenge, especially liver cancer, which is tricky, rare, and expensive [36] leading patients to seek treatment at an advanced stage when it is too late to cure them. Hence, policymakers must focus on low‐cost screening, diagnosis and awareness of Hepatitis B vaccination, the main contributors to liver cancer [37]. The authors also urge the government to pay attention to the breakthrough development that Bangladeshi scientists have made concerning early liver cancer detection through blood samples [35].

According to our study data, the extreme burden of cancer in its advanced stages was significantly higher than in its earlier stages. The extreme burden for patients with stage III and IV cancer was 39 and 25 times higher than those with stage 0 cancer, respectively. This result is consistent with previous studies showing that the economic burden of cancer tends to increase as cancer progresses to its advanced stages [38, 39]. For example, one study reported a higher economic burden at stage IV than at stage 0 [38], and another found that it was double in stage IV compared to stage III [39]. This high economic burden in advanced‐stage cancer may be due to the requirement of advanced care, aggressive treatments like chemotherapy, radiotherapy, surgery, and patients' critical condition. Earlier detection may offer more efficient treatment options, avoid complications and disease progression [35, 38, 39], therefore, the authors emphasize ensuring available cancer screening, treatment options, and cancer management facilities in Bangladesh.

Financial concerns affect patient‐reported outcomes, leading to lower patient satisfaction and worse quality of life [6, 7, 8, 40]. Evidently, a large number of households in LMICs, including Bangladesh incur financial debts and sell assets in order to finance their health care payments, especially for non‐communicable diseases like cancer and cardiovascular diseases [41]. We found that patients from low socio‐economic status (poorest income quintile: Q_1_) had a 9‐fold higher extreme economic burden compared to patients from wealthiest communities (richest income quintile: Q_5_). The findings show the serious consequences of the economic burden on cancer patients with poor financial status when they have higher levels of hardship healthcare financing than the rich due to OOP healthcare expenditure in Bangladesh [22]. Hence, the government should increase funding for health services such as healthcare subsidies, and financial assistance that promote health and poverty reduction to minimize the burden on patients and their families, particularly the poorest.

Our study findings offer insights for policymakers, clinicians, and researchers regarding the economic impact of cancer in Bangladesh. The findings can be used to advocate for scaling up cancer screening, early diagnosis, available treatment options, financial assistance and healthcare subsidies for patients to reduce the burden of the disease and improve patient outcomes. Our evidence on the high economic burden of advanced‐stage cancer provides proof of the economic benefits of cancer screening and early diagnosis [12]. The study revealed that patients with prostate, bone, and liver cancer face a high economic burden that raises concerns about screening, diagnosis, and affordable treatment options for these diseases. As a result, it is imperative to focus on multiple fronts, including awareness, cost‐effective approaches, health system strengthening, and public health policies to reduce cancer economic burden. Also, our results highlight the need for additional efforts to curb cancer burdens for socioeconomically disadvantaged patients by implementing health insurance, health care subsidies, and discounted or free treatment.

This study has some limitations. Firstly, the associated economic burden figures, including direct and indirect medical costs, societal costs, and productivity losses of patients and their families, are lacking in our study, which may influence the estimation. Further, our estimation could be impacted by the patient's condition, resulting in under‐ or over‐estimation. The nature of our study design may pose challenges to causality. Recall bias might occur when participants are asked to report the cost burden incurred over the past 3 months. Finally, this study is limited to a limited number of patients based on the capital city‐based hospitals. This may not be sufficient to generalize the whole population in Bangladesh. Despite these limitations, our study exhibits several strengths. First, our study explored the pattern and snapshot of economic burden across the cancer types, stages, and patients' socioeconomic conditions, which are helpful for generating hypotheses and further investigation. Face‐to‐face interviews with cancer patients at the country's most prominent cancer hospitals might help to generate an accurate conclusion for proper policy planning. Further, our study can provide baseline information for decision‐makers, researchers, and healthcare providers in planning interventions and proper resource allocation.

## 5 Conclusions

Our study found a disproportionately high economic burden among patients with cancer, across disease sites, stages, and income quintiles. The burden was significantly higher among patients with prostate, bone, and liver cancer, and those diagnosed with advanced cancer. The findings underscore the importance of early detection of cancer which may lead to more efficient treatment, avoiding disease progression, complications, lower disease management costs, and better health outcomes. Patients from low‐income households experience an extreme economic burden due to cancer, highlighting the need for affordable healthcare services, financial support, and healthcare subsidies. Further research is needed to explore the cost‐effective cancer care strategy to inform policies concerning health resource allocation decisions.

## Author Contributions

**Md. Shahjalal:** conceptualization, methodology, formal analysis, resources, project administration, writing – original draft, writing – review and editing, investigation, data curation, visualization, validation. **Padam Kanta Dahal:** writing – review and editing, visualization, validation, data curation, resources, investigation, project administration. **Md. Parvez Mosharaf:** software, formal analysis, data curation, validation, visualization, resources, writing – review and editing. **Mohammad Morshad Alam:** visualization, validation, writing – review and editing, project administration, resources. **Mohammad Delwer Hossain Hawlader:** writing – review and editing, visualization, validation, project administration. **Rashidul Alam Mahumud:** methodology, formal analysis, investigation, writing – review and editing, supervision, project administration, resources, visualization, validation.

## Ethics Statement

All procedures performed in studies involving human participants were in accordance with the 1964 Helsinki Declaration. The ethics committee of North South University, Bangladesh approved the study protocol (Ref‐2021/OR‐NSU/IRB/0401). For data collection in the hospitals, official permission was obtained from the respective authorities prior to the survey. All participants provided informed consent before participating in this study, and their details were de‐identified. Participants voluntarily participated in the study and had the right to withdraw from the study without a reason.

## Conflicts of Interest

The authors declare no conflicts of interest.

## Data Availability

The datasets that arose and were used in the current study are available from the corresponding author upon reasonable request.

## Appendix 2

**TABLE A1:** Magnitude of economic burden among cancer patients by gender.

Variables	Male						Female					
	Magnitude of economic burden of cancer											
	No/limited burden		Severe burden		Extreme burden		No/limited burden		Severe burden		Extreme burden	
	*n*	%	*n*	%	*n*	%	*n*	%	*n*	%	*n*	%
Cancer types												
Oral	14	15.22	10	10.53	19	19.79	7	6.80	4	3.33	13	11.11
Breast^a^	1	1.09	na	na	na	na	25	24.27	45	37.50	38	32.48
Lung	15	16.3	18	18.95	20	20.83	5	4.85	4	3.33	4	3.42
Blood	3	3.26	3	3.16	4	4.17	5	4.85	3	2.50	1	0.85
Gallbladder	2	2.17	na	na	1	1.04	2	1.94	3	2.50	1	0.85
Pancreatic	6	6.52	na	na	2	2.08	2	1.94	1	0.83	2	1.71
Liver	3	3.26	5	5.26	8	8.33	2	1.94	1	0.83	2	1.71
Kidney	4	4.35	1	1.05	na	na	na	na	1	0.83	1	0.85
Colorectal	2	2.17	7	7.37	5	5.21	4	3.88	4	3.33	9	7.69
Prostate	1	1.09	4	4.21	6	6.25	na	na	na	na	na	na
Urinary tract	2	2.17	4	4.21	1	1.04	1	0.97	1	0.83	na	na
Cervical	na	na	na	na	na	na	29	28.16	29	24.17	28	23.93
Brain	3	3.26	2	2.11	3	3.13	1	0.97	1	0.83	3	2.56
Gastric	3	3.26	na	na	2	2.08	1	0.97	2	1.67	1	0.85
Bone	2	2.17	2	2.11	4	4.17	1	0.97	1	0.83	6	5.13
Throat	6	6.52	9	9.47	1	1.04	2	1.94	1	0.83	2	1.71
Esophageal	na	na	5	5.26	2	2.08	1	0.97	1	0.83	na	na
Other cancers	25	27.17	25	26.32	18	18.75	15	14.56	18	15.00	6	5.13
Cancer stages												
Stage 0	5	5.43	5	5.26	2	2.08	8	7.77	na	na	na	na
Stage I	45	48.91	41	43.16	39	40.63	47	45.63	49	40.83	44	37.61
Stage II	30	32.61	29	30.53	29	30.21	29	28.16	40	33.33	45	38.46
Stage III	7	7.61	16	16.84	21	21.88	14	13.59	22	18.33	23	19.66
Stage IV	5	5.43	4	4.21	5	5.21	5	4.85	9	7.5	5	4.27

Abbreviation: na, not available.

^a^ One male patient had history of breast cancer.
